# Heparin stability in parenteral nutrition bags prepared in a neonatal ICU

**DOI:** 10.1186/cc13286

**Published:** 2014-03-17

**Authors:** A Foinard, M Perez, B Décaudin, C Barthélémy, A Tournoys, L Storme, P Odou

**Affiliations:** 1 Faculté de Pharmacie, Lille, France; 2 Institut de Pharmacie, CHRU, Lille, France; 3 Fédération de Biologie Pathologie du CHRU, Lille, France; 4 Hôpital Jeanne de Flandre, CHRU, Lille, France

## Introduction

Heparin is commonly given in our neonatal ICU (NICU) by continuous intravenous infusion. Heparin is diluted in parenteral nutrition bags and administered over a period of 24 hours with in-line filtration. However, there are no data on heparin stability in parenteral nutrition bags, especially on its compatibility with 50% dextrose mainly present in bags. The aim of our *in vitro *study was to determine heparin stability in parenteral nutrition bags prepared in a NICU after 24-hour infusion and to assess its interaction or not with 50% dextrose.

## Methods

We prepared both types of bag: parenteral nutrition bags whose composition was defined in the unit, including sodium heparin (77 UI/ml); and bags containing only sodium heparin diluted in 50% dextrose (193 UI/ml). These bags (*n *= 6 per type) were infused over a period of 24 hours with and without in-line filtration. Heparin activity was measured using a chromogenic anti-Xa method in bags just being prepared (references for other measures) and after 24-hour infusion and in effluents at the end of infusion line after 24 hours.

## Results

Our results show values of heparin activity measured in bags and effluents with and without in-line filtration after 24-hour infusion for both types of bag assessed (Tables 1 and 2). Results are expressed as median values (minimum to maximum) in percent.

**Table 1:** Results of heparin activity in parenteral nutrition bags

	Bags at T0	Bags at T0 + 24 hours	Effluent at T0 + 24 hours
With filtration	100 (84.09 to 109.09)	95.45 (79.55 to 102.27)	97.73 (75.00 to 104.55)
Without filtration	100 (90.48 to 114.29)	97.62 (83.33 to 107.14)	97.62 (83.33 to 114.29)

**Table 2:** Results of heparin activity in bags with sodium heparin in 50% dextrose

	Bags at T0	Bags at T0 + 24 hours	Effluent at T0 + 24 hours
With filtration	100 (77.45 to 108.82)	95.10 (90.20 to 109.80)	98.04 (95.10 to 100.98)
Without filtration	100 (85.44 to 107.77)	92.23 (88.35 to 100.00)	94.17 (86.41 to 108.74)

## Conclusion

We conclude that there is no loss of heparin activity when drug is infused over 24 hours for both types of bag prepared, with and without in-line filtration, showing heparin activity remains stable during this period and there is no interaction of drug with other nutrient components of bags, especially 50% dextrose.

